# In-Hospital Mortality and Prediction in an Urban U.S. Population With COVID-19

**DOI:** 10.7759/cureus.11786

**Published:** 2020-11-30

**Authors:** Vinod Rustgi, Michael Makar, Carlos D Minacapelli, Kapil Gupta, Abhishek Bhurwal, You Li, Carolyn Catalano, Reynold Panettieri

**Affiliations:** 1 Medicine, Division of Gastroenterology and Hepatology, Rutgers Robert Wood Johnson Medical School, New Brunswick, USA; 2 Medicine, Center for Liver Diseases and Liver Masses, Rutgers Robert Wood Johnson Medical School, New Brunswick, USA; 3 Medicine, Rutgers Robert Wood Johnson Medical School, New Brunswick, USA; 4 Medicine, Rutgers Institute for Translational Medicine and Science, New Brunswick, USA

**Keywords:** covid-19 infection, mortality, risk factors, score calculator

## Abstract

Coronavirus disease 2019 (COVID-19) has touched every aspect of society, and as the pandemic continues around the globe, many of the clinical factors that influence the disease course remain unclear. A useful clinical decision-making tool is a risk stratification model to determine in-hospital mortality as defined in this study. The study was performed at Robert Wood Johnson University Hospital (RWJUH) in New Brunswick, New Jersey, USA. Data was extracted from our electronic medical records on 44 variables that included demographic, clinical, laboratory tests, treatments, and mortality information. We used the least absolute shrinkage and selection operator regression with corrected Akaike’s information criterion to identify a subset of variables that yielded the smallest estimated prediction error for the risk of in-hospital mortality. During the study period, 808 COVID-19 patients were admitted to RWJUH. The sample size was limited to patients with at least one confirmed in-house positive nasopharyngeal swab COVID-19 test. Pregnant patients or those who were transferred to our facility were excluded. Patients who were in observation and were discharged from the emergency room were also excluded. A total of 403 patients had complete values for all variables and were eligible for the study. We identified significant clinical, laboratory, and radiologic variables determining severe outcomes and mortality. An in-hospital mortality risk calculator was created after the identification of significant factors for the specific cohort, which were abnormal CT scan or chest X-ray, chronic kidney disease, age, white blood cell count, platelet count, alanine aminotransferase, and aspartate transaminase with a sensitivity, specificity, and negative predictive value of 82%, 72%, and 93%, respectively. While numerous reports from around the globe have helped outline the pandemic, demographic factors vary widely. This study is more applicable to an urban, highly diverse population in the United States.

## Introduction

The pandemic caused by the severe acute respiratory syndrome coronavirus 2 (SARS-CoV-2) [1], previously known as the 2019 novel coronavirus (2019-nCoV) [2], has wreaked havoc. Clinicians, scientists, data scientists, vaccine experts, public policy specialists, and others, as well as the highest levels of governments globally are focused on COVID-19 (coronavirus disease 2019) [3] as it has touched every aspect of society.

Coronaviruses are enveloped, positive-sense single-stranded RNA viruses that are classified together on the basis of the crown or halo-like appearance of the spike envelope glycoproteins [4]. The name is derived from the Latin word corona, which means crown. To date, seven human coronaviruses have been identified, and based on the published information, SARS-CoV-2 is the third zoonotic human coronavirus of the century [5]. This new agent causes symptoms ranging from a dry cough to dyspnea to a syndrome with protean manifestations including severe respiratory distress, thrombotic conditions, and other clinical problems that are still being identified [6,7].

While the first cases of COVID-19 were reported from Wuhan, China [6], it was identified in the United States (US) by the end of January 2020, initially in Washington State [8]. By the end of July of 2020, there were more than four million cases in the US with over 150,000 fatalities [9]. As the pandemic continues around the globe, many of the clinical factors that influence the disease course remain unclear. In addition, the recently available research associated with risk factors and disease severity comes from centers that lack ethnic and racial diversity [10,11]. Understanding the clinical risk factors from multi-ethnic populations to determine disease severity and outcomes is needed to improve patient management.

Early identification of hospitalized COVID-19 patients at higher risk of mortality may help ensure proper clinical care and increased survival. Liang et al. developed a clinical risk score to predict critical illness in patients hospitalized with COVID-19 [12]. While the score was developed using data on 1,590 Chinese patients, the average age of admitted patients was 48.9 years and an estimated 74.9% of all hospitalized patients reported no comorbidities. In contrast, the average age of hospitalized COVID-19 patients in the US is 61 years, with an estimated hypertension prevalence of 43.5% [13]. Hence, the risk score developed by Liang et al. may not fully represent the clinical experience of hospitalized patients in the US.

To address the research gap, this study analyzed a racially and ethnically diverse adult, inpatient, laboratory-confirmed COVID-19 population at Rutgers Robert Wood Johnson University Hospital (RWJUH), a 965-bed University hospital in New Brunswick, New Jersey, USA. The patient population at RWJUH is more representative of urban areas in the US. Abstracted laboratory, demographic, and clinical data that were found to be significant were used to develop a risk stratification model to determine mortality risk.

## Materials and methods

Data source and sample selection

**Figure 1:**
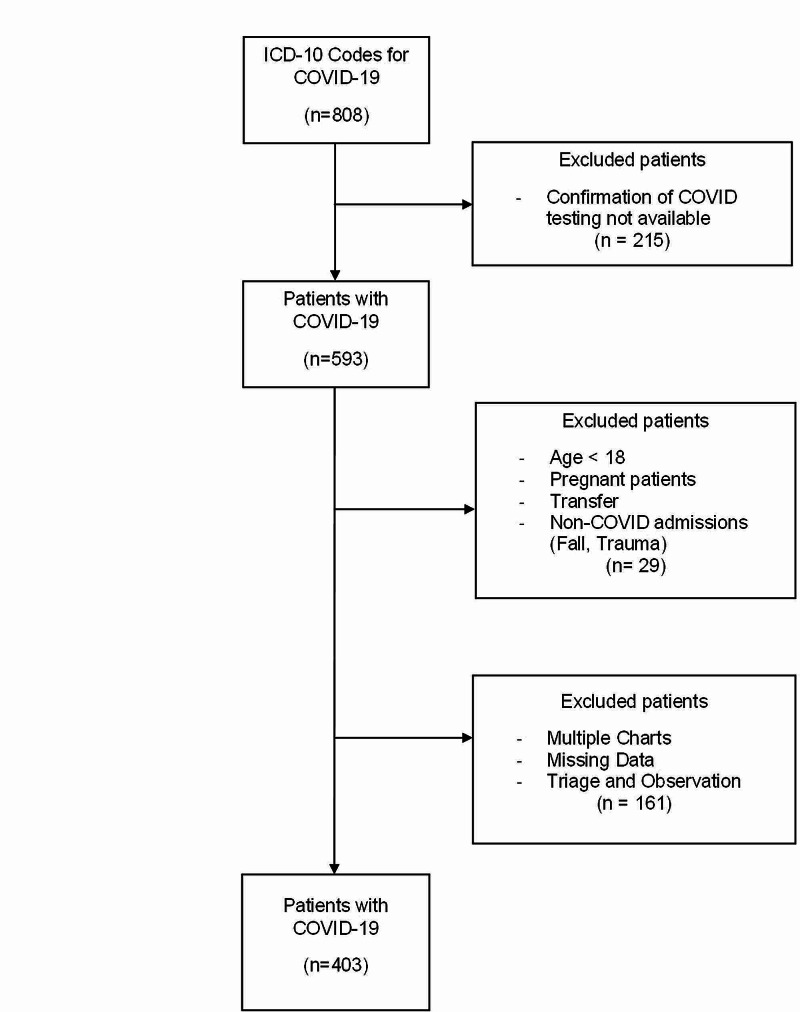
Study Cohort Inclusion/Exclusion Criteria

We conducted a cohort study of COVID-19 patients using RWJUH electronic medical records (EMR) under an IRB-approved protocol. The study included all adult (≥18 years old) COVID-19 patients who were admitted to RWJUH between January 1, 2020, and April 30, 2020. As per the Centers for Disease Control (CDC) guidelines, we identified COVID-19 cases using our EMR with the International Classification of Disease, 10th Revision, Clinical Modification (ICD-10-CM) code B97.29 for hospital discharges between January 1, 2020, and March 31, 2020, and ICD-10-CM code U07.1 for discharges that took place thereafter (n=808). Sample size was limited to patients with at least one confirmed positive nasopharyngeal swab SARS-CoV-2 test at our facility (n=593). Subsequently, pregnant patients or those who were transferred were excluded. The study excluded 72 patients who were under observation and were never admitted and an additional 45 patients who were discharged from the emergency room. A total of 403 patients with a confirmed SARS-CoV-2 test and a complete data set with variables of interest who were admitted to RWJUH inpatient services were identified (Figure 1).

Study variables

Data were extracted manually from the EMR (SCM, Allscripts) and included demographic, clinical, laboratory, radiological, in-hospital treatments, and mortality data. Relevant comorbidities were identified. Medication lists at admission were reconciled.

All data were checked and reviewed by three different physician reviewers. The study’s main outcome was in-hospital mortality for patients admitted with COVID-19. All mortality data were acquired from the EMR and were confirmed through medical chart reviews. A total of 44 variables were considered for the predictive model, including data on patients’ demographics, clinical characteristics, imaging findings, and laboratory results that were collected at admission. Patients’ demographics included gender, race/ethnicity, and age. Clinical characteristics considered for the predictive model included body mass index (BMI) and presenting symptoms such as fever, cough, dyspnea, anosmia, diarrhea, nausea, emesis, anorexia, malaise, and altered mental status (AMS). Prior medication use including proton-pump inhibitors (PPIs), nonsteroidal anti-inflammatory drugs (NSAIDs), angiotensin-converting enzyme (ACE) or angiotensin II receptor blocker (ARB) inhibitor, insulin or oral hypoglycemics, oral steroids, calcium channel blockers (CCB), statins, or beta-blockers were incorporated.

Patients’ medical histories, which included the total number and type of comorbidities, co-infections, and hospital readmission status within 30 days prior to current admission, were gathered (Table 1). Imaging findings included results from both chest X-rays (CXRs) and computed tomography (CT) scans. We considered the following laboratory findings: white blood cells (WBCs), mean corpuscular volume (MCV), platelet count, blood urea nitrogen (BUN), creatinine (Cr), bicarbonate, albumin, total bilirubin (T Bili), alanine aminotransferase (ALT), and aspartate transaminase (AST). We also reviewed results of neutrophil, lymphocytes, D-dimer, C-reactive protein (CRP), and electrocardiography. However, those variables were not considered for the predictive model due to high proportions of patients with missing values.

**Table 1:** Sample Characteristics by In-Hospital Mortality Status for Patients Admitted With COVID-19 (n=403) ^a^The comparisons between those with versus without in-hospital mortality. ^b^Six patients with in-hospital mortality had missing BMI values. ^c^Including those with poor appetite or PO intake. ^d^Including ulcerative colitis, Crohn's disease, gastroesophageal reflux disease, viral hepatitis, irritable bowel syndrome, cirrhosis, and alcoholic hepatitis. SD, standard deviation; BMI, body mass index; AMS, altered mental status; PPI, proton-pump inhibitors; NSAIDS, nonsteroidal anti-inflammatory drugs; ACE, angiotensin-converting enzyme; ARB, angiotensin II receptor blockers, CCB, calcium channel blockers; MI, myocardial infarction; CAD, coronary artery disease; COPD, chronic obstructive pulmonary disease, GI, gastrointestinal; ICU, intensive care unit

Characteristic	In-Hospital Mortality		p-Value^a^
	No (n=313)	Yes (n=90)	
Age, mean (SD) [range]	58.8 (18.3) [45.0-73.0]	73.4 (15.0) [64.0-85.0]	<0.0001
Gender, n (%)			
Male	176 (56.2)	56 (62.2)	0.3107
Female	137 (43.8)	34 (37.8)	
Race, n (%)			
White, non-Hispanics	90 (28.8)	42 (46.7)	0.0003
Black, non-Hispanics	37 (11.8)	7 (7.8)	
Hispanics	120 (38.3)	14 (15.6)	
Asian	39 (12.5)	15 (16.7)	
Other	27 (8.6)	12 (13.3)	
BMI^b ^(kg/m^2^), mean (SD)	28.78 (6.5)	28.94 (9.3)	0.8788
Presenting symptoms, n (%)			
Fever	227 (72.5)	64 (71.1)	0.7920
Cough	197 (62.9)	50 (55.6)	0.2050
Dyspnea	223 (71.3)	59 (65.6)	0.2993
Anosmia	7 (2.2)	1 (1.1)	0.6902
Diarrhea	71 (22.6)	9 (10.0)	0.0078
Nausea	60 (19.2)	7 (7.8)	0.0105
Emesis	43 (13.7)	8 (8.9)	0.2811
Anorexia^c^	59 (18.9)	12 (13.3)	0.2260
Malaise	77 (24.6)	15 (16.7)	0.1140
AMS	37 (11.8)	31 (34.4)	<0.0001
Prior medication use, n (%)			
PPI	57 (18.2)	24 (26.7)	0.0777
NSAIDS	12 (3.8)	9 (10.0)	0.0204
ACE/ARB inhibitor	86 (27.5)	29 (32.2)	0.3796
Insulin or oral hypoglycemic	100 (31.9)	33 (36.7)	0.4016
Oral steroid	16 (5.1)	5 (5.6)	0.7931
CCB	64 (20.5)	24 (26.7)	0.2082
Statins	95 (30.4)	38 (42.2)	0.0348
Beta-blocker	76 (24.3)	24 (26.7)	0.6443
No. of comorbidities, n (%)			
Chronic kidney disease	210 (67.1)	79 (87.8)	0.0001
Malignancy	21 (6.7)	15 (16.7)	0.0035
Hypertension	174 (55.6)	63 (70.0)	0.0144
Hyperlipidemia	99 (31.4)	35 (38.9)	0.1976
Type 2 diabetes	116 (37.1)	38 (42.2)	0.3745
Congestive heart failure	18 (5.8)	11 (12.2)	0.0363
MI/CAD	41 (13.1)	22 (24.4)	0.0090
Dementia	22 (7.0)	18 (20.0)	0.0003
Peptic ulcer	1 (0.3)	1 (1.1)	0.3972
Mild liver disease	2 (0.6)	1 (1.1)	0.5325
Peripheral vascular disease	12 (3.8)	4 (4.4)	0.7938
Cerebrovascular disease	18 (5.6)	19 (21.1)	<0.0001
Hemiplegia/paraplegia	3 (1.0)	3 (3.3)	0.1280
Asthma	21 (6.7)	4 (4.4)	0.6202
HIV/AIDS	4 (1.3)	0 (0.0)	0.5792
Hypothyroid disease	24 (7.7)	6 (6.7)	0.7498
Rheumatoid arthritis	8 (2.6)	2 (2.2)	1.0000
Depression	14 (4.5)	8 (8.9)	0.1160
Seizures	4 (1.3)	5 (5.6)	0.0295
COPD	17 (5.4)	11 (12.2)	0.0256
GI-specific comorbidities,^d^ n (%)	44 (14.1)	15 (16.7)	0.5372
Co-infections, n (%)	24 (7.7)	13 (14.4)	0.0497
Readmission within past 30 days, n (%)	69 (22.0)	13 (14.4)	0.1145
Length of hospital stay, mean (SD) [range]	5.70 (4.0) [3.0-7.0]	7.2 (4.7) [4.0-9.0]	0.0086
Admission to ICU, n (%)	35 (11.2)	53 (58.9)	<0.0001
Length of ICU stay, mean (SD) [range]	5.26 (4.0) [3.0-7.0]	6.26 (4.7) [3.0-9.0]	0.2969
Mechanical ventilation, n (%)	23 (7.4)	49 (54.4)	<0.0001
Abnormal computed tomography or chest X-ray, n (%)	274 (87.5)	83 (92.2)	0.2183

Statistical analysis

Patient and hospitalization characteristics for those with versus those without in-hospital mortality were represented as numbers and percentages for categorical variables and as means and standard deviations for continuous variables. Chi-square and Student’s t-tests were used to characterize the study sample according to mortality status. We quantified both crude and race, gender, and age-adjusted means for all laboratory findings by mortality status.

All patients with non-missing values were included in the development of the in-hospital mortality prediction model. Least absolute shrinkage and selection operator (LASSO) regression for variable selection and predictive model construction were utilized. The LASSO method aims to constrain the regression coefficients by shrinking their value towards zero using a shrinkage parameter. In LASSO, the shrinkage parameter λ is imposed on the sum of absolute values of the regression coefficients L1 norm. As λ increases, the values of the regression coefficients shrink toward zero. We used the LASSO regression with corrected Akaike’s information criterion to identify a subset of the 44 study variables that yields the smallest estimated prediction error for the risk of in-hospital mortality. In turn, the identified variables were included in logistic regression models to determine the subset of predictive variables that were statistically significant. The accuracy of the predictive model was evaluated using the area under the receiver operating characteristic curves (AUC). The final set of predictive variables was used to estimate the probability of in-hospital mortality. The optimal cut-off point was then determined to classify COVID-19 patients as with or without high risk of in-hospital mortality. To estimate this optimal cut-off point, the closest point on receiver operating curve (ROC) to the ideal prediction point was used (i.e., where sensitivity = 1 and specificity = 0).

Sensitivity analysis

The predictive model AUC accuracy was examined using the leave-one-out cross-validation method. To determine how frequently each of the 44 variables is selected, 10,000 bootstrap resamples LASSO regression with the Schwarz Bayesian Information Criterion (SBC) for variable selection were used. In turn, we examined the estimated selection frequency of the predictive variables selected in the final model as a measure of effect importance. A significance level of 0.05 for two-sided tests was considered statistically significant. All 95% confidence intervals (CIs) were reported when applicable. All analyses were performed using SAS 9.4 software (SAS Institute, Cary, NC, USA).

## Results

Sample characteristics

Of the COVID-19 patients admitted to RWJUH during the study period, 403 had non-missing values on any of the variables used in the predictive model selection process (Figure 1). Patient characteristics by in-hospital mortality status are summarized in Table 1. The overall mortality in our sample was 22.3%. Compared to those without, patients with in-hospital mortality were older (mean age 58.8 years vs. 73.4 years; p<0.0001). Race was significantly associated with in-hospital mortality status. Patients with in-hospital mortality were more likely to report AMS (34.4% vs. 11.82%; p<0.0001) and less likely to report both diarrhea (10.0% vs. 22.7%; p=0.0078) and nausea (19.2% vs. 7.8%; p=0.0105) than those who were discharged alive. Higher proportions of patients with in-hospital mortality reported using NSAIDs and statins.

At admission, patients with in-hospital mortality were more likely to have a history of chronic kidney disease (CKD), malignancy, hypertension, congestive heart failure (CHF), MI (myocardial infarction)/CAD (coronary artery disease), dementia, cerebrovascular disease, seizures, and chronic obstructive pulmonary disease (COPD). The average length of stay was 1.5 days longer for those with versus those without in-hospital mortality. Patients who died during their hospital stay were more likely to be admitted to the ICU and be placed on mechanical ventilation than those who were discharged alive.

Patients’ laboratory findings by mortality status are shown in Table 2. Patients with in-hospital mortality were generally characterized with abnormal laboratory results. Specifically, higher average values were seen for BUN (33.1 mg/dL vs. 20.6 mg/dL; p<0.0001), T Bili (0.62 mg/dL vs. 0.51 mg/dL; p=0.0305), and CRP (14.7 mg/dL vs. 12.0 mg/dL; p=0.023) for those with, relative to those without, in-hospital mortality. In contrast, platelet count and albumin were significantly lower in those who died in-hospital than patients who were discharged alive. After adjustment for age, gender, and race, values for platelet count, BUN, albumin, and CRP remained significantly different between the two groups.

**Table 2:** Crude and Adjusted Laboratory Findings by In-Hospital Mortality Status for Patients Admitted With COVID-19 (n=403) ^a^Adjusted for race, gender, and age. ^b^From t-test for the comparisons between those with versus without in-hospital mortality. ^c^From linear regression for the comparisons between those with versus without in-hospital mortality. Note: Data are presented as mean (SD)

Variable	Number of Patients	Unadjusted			Adjusted^a^		
		In-Hospital Mortality		p-Value^b^	In-Hospital Mortality		p-Value^c^
		No (n=313)	Yes (n=90)		No (n=313)	Yes (n=90)	
White blood cells (thousand/uL)	419	8.58 (0.25)	9.19 (0.56)	0.3208	8.64 (0.30)	9.61 (0.54)	0.1066
Mean corpuscular volume (fL)	419	86.60 (0.41)	88.13 (0.85)	0.0912	86.24 (0.48)	86.63 (0.86)	0.6797
Platelet count (thousand/uL)	419	241.35 (5.56)	200.92 (7.70)	<0.0001	244.44 (6.08)	206.79 (11.00)	0.0022
Neutrophil (thousand/uL)	351	8.23 (0.66)	9.58 (1.50)	0.4111	8.12 (0.80)	9.37 (1.44)	0.4349
Lymphocyte (thousand/uL)	351	1.27 (0.12)	1.25 (0.29)	0.9500	1.17 (0.15)	1.16 (0.26)	0.9952
D-dimer level (ng/mL)	387	3176.41 (558.75)	5839.03 (2169.93)	0.2376	3281.94 (855.13)	5237.98 (1502.15)	0.2455
Blood urea nitrogen (mg/dL)	414	20.58 (1.10)	33.31 (2.87)	<0.0001	23.18 (1.28)	28.89 (2.30)	0.0258
Creatinine (mg/dL)	414	1.30 (0.09)	1.74 (0.23)	0.0724	1.41 (0.11)	1.67 (0.20)	0.2530
Bicarbonate (mmol/L)	413	21.82 (0.19)	21.57 (0.34)	0.5243	21.77 (0.21)	21.44 (0.38)	0.4176
Albumin (g/dL)	404	3.68 (0.09)	3.29 (0.07)	0.0006	3.36 (0.09)	3.27 (0.17)	0.0410
Total bilirubin (mg/dL)	403	0.51 (0.02)	0.62 (0.05)	0.0305	0.53 (0.02)	0.60 (0.04)	0.0860
Alkaline phosphatase (IU/L)	403	84.85 (3.17)	77.81 (3.42)	0.1329	83.49 (3.27)	79.27 (5.86)	0.5189
Alanine aminotransferase (IU/L)	403	50.18 (4.55)	46.27 (6.24)	0.6128	49.27 (4.90)	52.05 (8.74)	0.7765
Aspartate transaminase (IU/L)	403	60.79 (5.00)	80.13 (8.80)	0.0651	63.90 (5.64)	84.72 (10.09)	0.0651
C-reactive protein (mg/dL)	375	12.04 (0.53)	14.68 (1.05)	0.0230	12.23 (0.61)	15.82 (1.10)	0.0035
Electrocardiography (QTc)	375	454.40 (14.07)	454.82 (7.56)	0.9793	448.47 (14.39)	433.63 (25.86)	0.6059

Predictive model selection

A total of 44 variables were included in the model selection process using LASSO regression. The LASSO regression selected 21 variables using corrected Akaike’s information criterion. Variables selected for predicting in-hospital mortality included gender, race, age, co-infections, readmission within the past 30 days, abnormal CT scan or CXR, medical history including CKD, malignancy, CHF, cerebrovascular disease, dementia, prior PPI, NSAID, or beta-blocker use, WBCs, platelet count, albumin, T Bili, ALT, and AST. Using the 21 LASSO selected variables yielded an AUC of 0.85 (95% CI: 0.81-0.89) for predicting in-hospital mortality.

**Figure 2:**
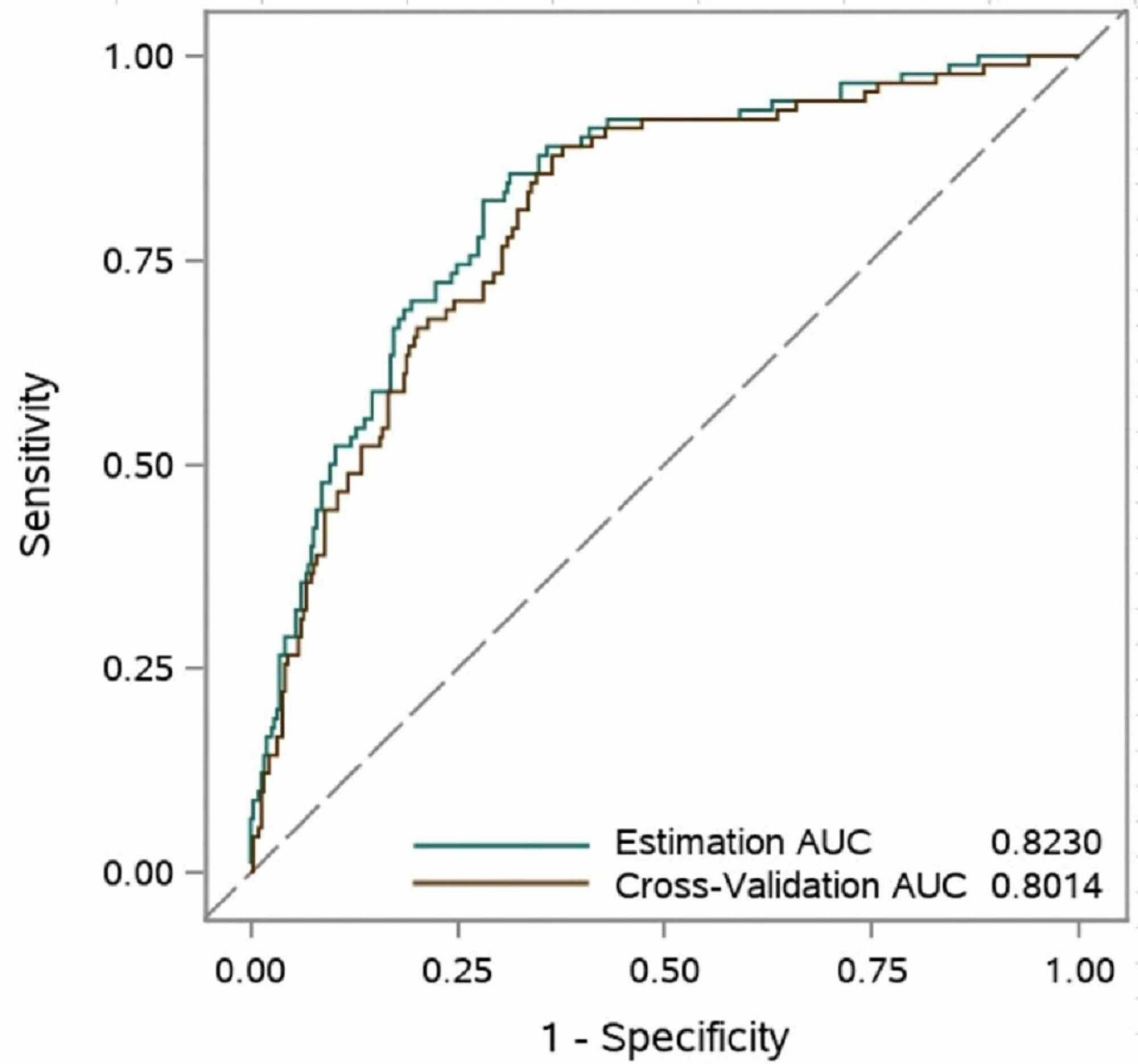
Receiver Operating Characteristic Curves of the Predictive Model in the Estimation Group (n=403) and Using the Leave-one-out Cross-Validation. The model included seven variables (abnormal CT or chest X-ray, chronic kidney disease, age, white blood cells, platelet count, alanine aminotransferase, and aspartate transaminase) to predict in-hospital mortality. The area under the ROC curves for the estimation and cross-validation analyses were 82.30 (95% CI: 77.52-87.09) and 80.14 (95% CI: 75.09-85.19), respectively.

Of the 21 variables selected by the LASSO method, only abnormal CT scan or CXR, CKD, age, WBCs, platelet count, ALT, and AST remained significant predictors of COVID-19 in-hospital morality using logistic regression models (Table 3). As a result, these seven variables were included in the COVID-19 in-hospital mortality prediction model. The AUC for predicting in-hospital mortality using these seven variables was 0.82 (95% CI: 0.78-0.87) (Figure 2).

**Table 3:** Multivariable Logistic Regression Model for Predicting In-Hospital Mortality for Patients Admitted With COVID-19 (n=403)

Variable	Model Coefficients	Multivariate Adjusted	p-Value
		Odds Ratio (95% CI)	
Constant	-5.5237		
Abnormal CT or chest X-ray (yes vs. no)	1.2274	3.41 (1.25-9.31)	0.0165
Chronic kidney disease (yes vs. no)	0.8752	2.40 (1.14-5.04)	0.0208
Age (years)	0.0417	1.04 (1.03-1.06)	<0.0001
White blood cells (per thousand/uL)	0.0879	1.09 (1.03-1.16)	0.0039
Platelet count (per thousand/uL)	-0.00643	0.994 (0.990-0.997)	0.0008
Alanine aminotransferase (per one IU/L)	-0.0246	0.976 (0.963-0.988)	0.0002
Aspartate transaminase (per one IU/L)	0.0217	1.02 (1.01-1.03)	<0.0001

COVID-19 In-Hospital Mortality Risk Score

**Figure 3:**
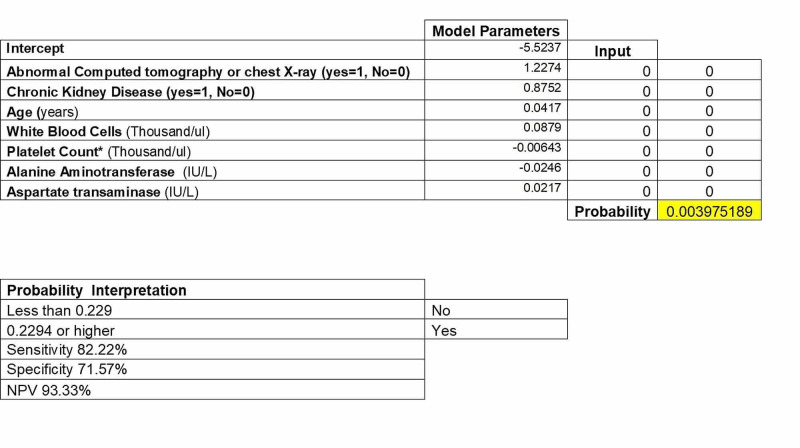
In-Hospital Mortality Risk Calculator

The probability of COVID-19 in-hospital mortality is equal to eX / (1 + eX) where X is [1.2274 * abnormal CT scan or CXR (yes=1 no=0)] + [(0.8752 * CKD) (yes=1 no=0)] + [0.0417 *age (years)] + [0.0879 * white blood cells (per thousand/ul)] - [ 0.00643 * platelet count per (Thousand/uL)] - [0.0246 * ALT (per IU/L)] + [0.0217 * AST (per IU/L)] - 5.5237. Using the shortest distance between the ROC and the ideal prediction point, the optimal cut-off for the probability of COVID-19 in-hospital mortality was 0.229. At this cut-off point, the model has a sensitivity, specificity, and negative predictive value of 82%, 72%, and 93%, respectively (Figure 3).

Sensitivity analysis

Using the leave-one-out cross-validation method, the AUC for predicting in-hospital mortality was 0.80 (95% CI: 0.75-0.85). Results from the 10,000 bootstrap resamples LASSO regression with the SBC for variable selection were consistent with our variables selection. As such, age was selected in 90.8% of the LASSO identified models, whereas abnormal CT scan or CXR, CKD, WBCs, platelet count, ALT, and AST were selected 50.0%, 58.3%, 46.6%, 90.2%, 72.5%, and 90.2%, respectively.

## Discussion

COVID-19 has rapidly become a leading focus of medical care in the United States and globally. A study from New York City of 1,150 hospitalized adults, of whom 257 (22%) were critically ill, showed older age, chronic cardiac disease, COPD, higher serum levels of interleukin-6, and D-dimer to be associated independently with mortality [14]. A meta-analysis from China of 8,697 patients showed the most commonly experienced symptoms were fever and cough [15]. The International Severe Acute Respiratory and Emerging Infections Consortium World Health Organization (ISARIC WHO) Clinical Characterization Protocol of 20,133 patients in the United Kingdom showed a four-day median duration between onset of symptoms and hospitalization [16]. In this study, the most common comorbidities were chronic cardiac disease, uncomplicated diabetes, non-asthmatic chronic pulmonary disease, and CKD. Independent risk factors for mortality were increasing age, male sex, and obesity. Older males may have a higher case fatality rate than females, perhaps due to differential expression of ACE2 receptors and TMPRSS2, a serine protease needed for spike protein priming [17]. Additional important factors may be sex hormone-driven innate and adaptive immune responses and immunoaging [18].

There is mounting evidence that the GI tract and the liver are also targets for viral entry [19]. The ACE2 receptor has been confirmed as an entry receptor [20]. The spike glycoprotein (S-protein) is instrumental in virus attachment and receptor recognition [21]. ACE2 receptors have are expressed in multiple areas of the GI tract, including the esophagus, ileum, and colon, as well as cholangiocyte [22]. In addition to nausea, vomiting, anorexia, and diarrhea as manifestations of GI involvement, liver enzyme abnormalities have been noted frequently [23]. Interestingly, our study found that GI symptoms of diarrhea and nausea had an inverse relationship with mortality (Table 1).

There have been variations in some of the findings reported in the literature. Mortality rates may be affected by variations in national healthcare delivery systems [24,25], variations in inpatient population demographic factors such as race and ethnicity [26], and variations in socioeconomic conditions [27]. The US has a multi-ethnic, multi-racial population in most large urban areas. This study offers further information in this context.

Our study reflected demographic factors more characteristic of the diversity of urban USA (Table 1). An estimated 33% of the patients admitted were Hispanic, 32.8% were White, 13.4% were Asian, and 10.9% were African-American; 62% were male and 38% were female. Risk factors affecting mortality included older age and AMS, whereas there was an inverse relationship with presenting symptoms of diarrhea or nausea. In our cohort, those who died had a significantly higher use of NSAIDs (10% vs. 3.84%, p=0.024) and statins (42.2% vs. 30.4%, p=0.035). Many studies have reported PPI [28,29] use as a risk factor, and although our data show a similar trend, it did not reach statistical significance.

Important comorbidities increasing risk of mortality included CKD, history of malignancy, hypertension, CHF, and MI/CAD, as well as the presence of dementia, cerebrovascular disease, history of seizures, and presence of COPD. We did not find diabetes to be an increased risk factor. Some of these findings may have been reflective of our patient population; i.e., the mean age of Caucasians who died was significantly higher than that of Hispanics and African-Americans and, therefore, perhaps reflected increased comorbidity burden. Interestingly, BMI seemed not to be a factor in mortality risk. Understandably, ICU admission, mechanical ventilation, and length of stay were correlated with mortality (Table 1).

As shown in Table 2, when adjusted for race, gender, and age, initial mean laboratory values of lower platelet counts (206,790 vs. 244,440/uL), slightly higher BUN values (28.9 vs. 23.2 mg/dL), lower albumin (3.27 vs. 3.36 g/dL), and higher CRP levels (15.82 vs 12.23 mg/dL) were predictive of in-hospital mortality. Liver enzymes, D-dimer levels, and QTc intervals on EKG were not differentiating.

From the analyses, a multivariable logistic regression model for predicting in-hospital mortality was developed with variables and model coefficients (Table 3). Additionally, a score calculator was developed (Figure 3). Seven variables in the model that were utilized were abnormal CXR or CT findings (yes or no), CKD (yes or no), age in years, WBCs, platelet count, ALT, and AST. The area under the ROC curves for the estimation and cross-validation analyses were 82.30 (95% CI: 77.52-87.09) and 80.14 (95% CI: 75.09-85.19), respectively. A probability calculation <0.229 or >0.2294 gave a sensitivity of 82.22% sensitivity and specificity of 71.57% for mortality with a negative predictive value of 93.33%. This in-hospital mortality risk calculator may allow quick decisions to be made, as to who is at risk of in-hospital mortality with a high degree of certainty, as well as those who may be sent home with close monitoring and communication. These are vital decisions, especially in the context of healthcare capacity constraints.

This study has limitations due to the relatively small sample size and its retrospective nature. Follow-up data on outpatients and their recovery were not available as the cohort was limited to inpatients. There may be an underestimate of mortality due to the limited time period studied. However, the strengths include information on a diverse patient population reflective of urban hospitals in the US, in distinction to many other parts of the world, and, therefore, generalizability.

The analyses in this paper add to the factors that help define in-hospital mortality based on initial laboratory values and comorbidities. These factors include increasing age, presenting symptoms of diarrhea and nausea, NSAID and statin use, AMS, CKD, history of malignancy, COPD, dementia and cerebrovascular disease, and history of seizures. Laboratory values of concern include lower admission platelet counts and lower albumin values, as well as higher BUN and CRP levels.

## Conclusions

The treatment for COVID-19 is rapidly evolving, and the mortality rates and outpatient care will improve accordingly. Simple medications such as dexamethasone are already making a difference. The data in this case-control study are helpful in defining the epidemiology of this pandemic. Furthermore, the calculator in this article may be of benefit in the interim in terms of triage decisions. This is of vital importance, as has been mentioned, due to capacity constraints and the resulting ethical implications and decisions.
